# Supplementary material for: Chlorine Modulation Fluorescent Performance of Seaweed-Derived Graphene Quantum Dots for Long-Wavelength Excitation Cell-Imaging Application
Source: Molecules. 2021 Aug 18;26(16):4994. doi: 10.3390/molecules26164994 (PMC8400823; doi:10.3390/molecules26164994)
Supplement: Supplementary file 1 [file molecules-26-04994-s001.zip › molecules-1304376-supplementary.pdf]

Communication

Chlorine Modulation Fluorescent Performance of Seaweed-Derived Graphene Quantum Dots for Long-Wavelength Excitation Cell-Imaging Application

Weitao Li [1,2,*], Ningjia Jiang [1], Bin Wu [3,*], Yuan Liu [4], Luoman Zhang [1], and Jianxin He [1,*]

1 Textile and Garment Industry of Research Institute, Zhongyuan University of Technology, Zhengzhou 450007, P.R. China; liweitao@zut.edu.cn (W.L.); m13470061821@163.com (N.J.); z1543086543@163.com (L.Z.); hejianxin771117@163.com (J.H.)

2 Institute of Nanochemistry and Nanobiology, School of Environmental and Chemical Engineering, Shanghai University, Shanghai 200444, P.R. China; liweitao@zut.edu.cn (W.L.)

3 School of Life Science and Technology, Tongji University, Shanghai 200082, P.R. China; tyronwb@163.com (B.W.)

4 Anhui Institute of Metrology, Anhui 230051, P.R. China; 18801911141@163.com (Y.L.)

* Correspondence: liweitao@zut.edu.cn (W.L.); tyronwb@163.com (B.W.); hejianxin771117@163.com (J.H.)

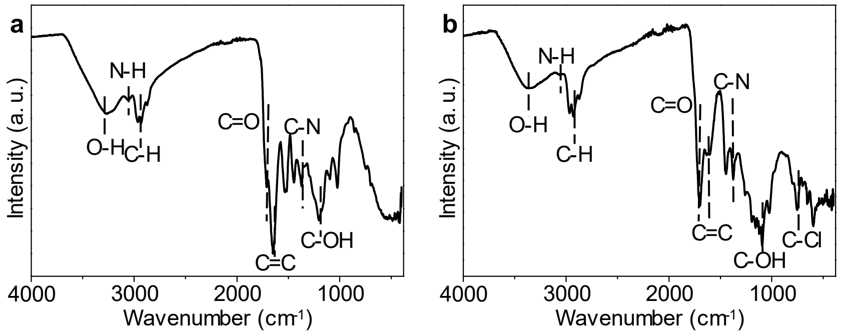

Figure S1. FT-IR spectra of (a) GQDs and (b) Cl-GQDs.

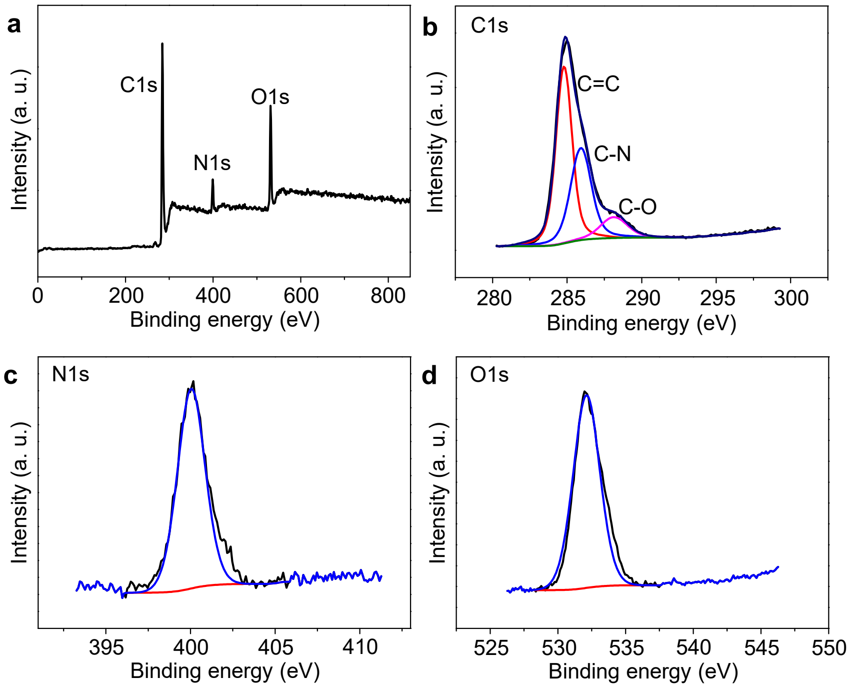

Figure S2. Structure Characterization of GQDs: (a) XPS survey spectrum, (b) C1s, (c) N1s, (d) O1s.

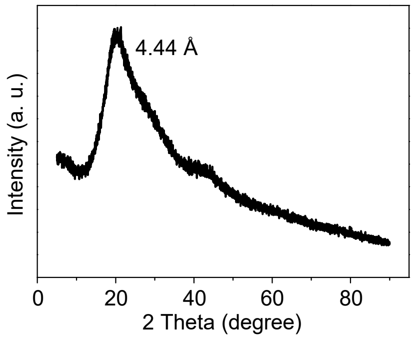

Figure S3. XRD patterns of GQDs.

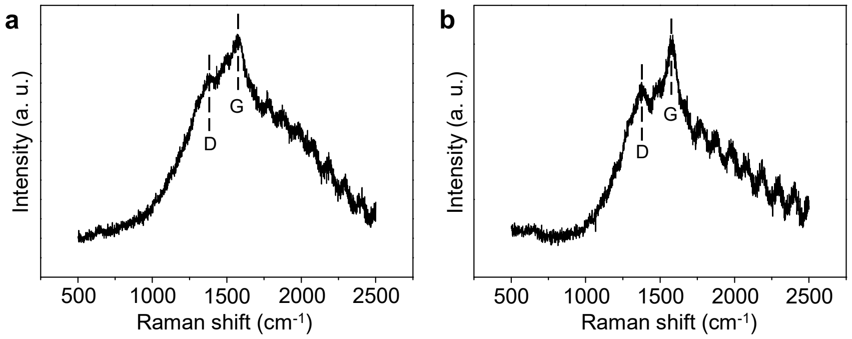

Figure S4. The typical Raman spectra of (a) GQDs and (b) Cl-GQDs.

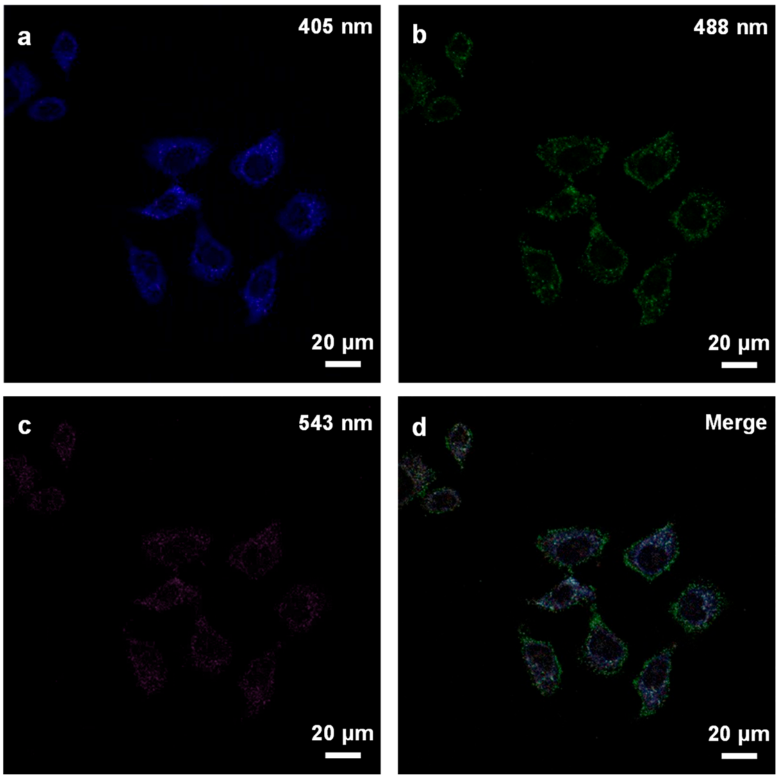

Figure S5. Cell-imaging of GQDs using HeLa cells excited at (a) 405 nm, (b) 488 nm, (c) 543 nm and (d) merged image.

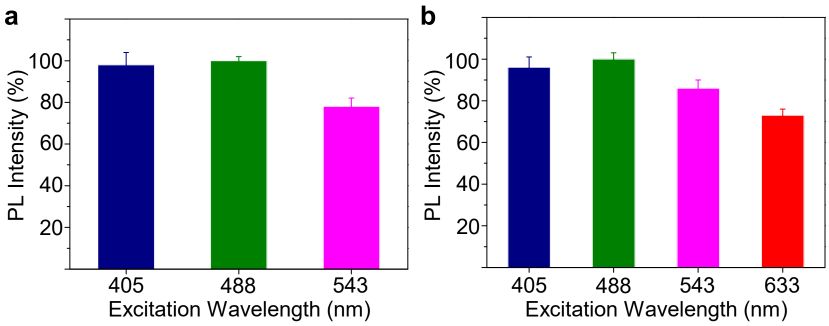

Figure S6. Fluorescence intensity analysis of HeLa cells at 405, 488, 543 and 633 nm by Image J: (a) GQDs and (b) Cl-GQDs.

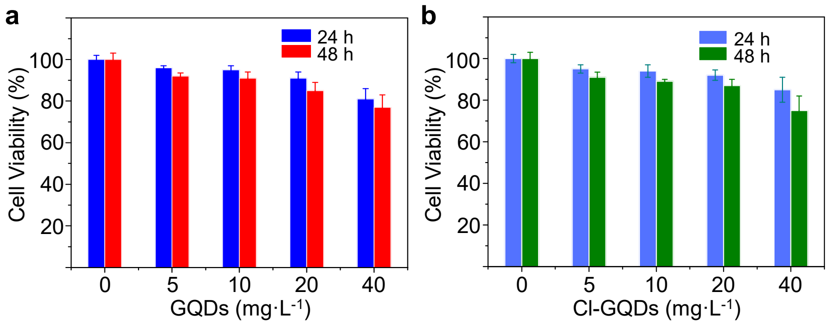

Figure S7. Cytotoxicity assessment of (a) GQDs and (b) Cl-GQDs at the imaging dose (5 mg·L^{-1}) and higher doses for incubation time varied from 24 to 48 h using HeLa cells. (3 measurements per group)

Table S1. The elements ratio of different Cl-doping GQDs in XPS survey spectra.

TCM ratio \ Elements	Cl (%)	C (%)	O (%)	N (%)
0%	0	76.10	15.96	7.94
10%	0.50	74.00	17.05	8.45
30%	2.38	74.42	19.03	4.17
50%	2.31	74.63	15.56	7.50
